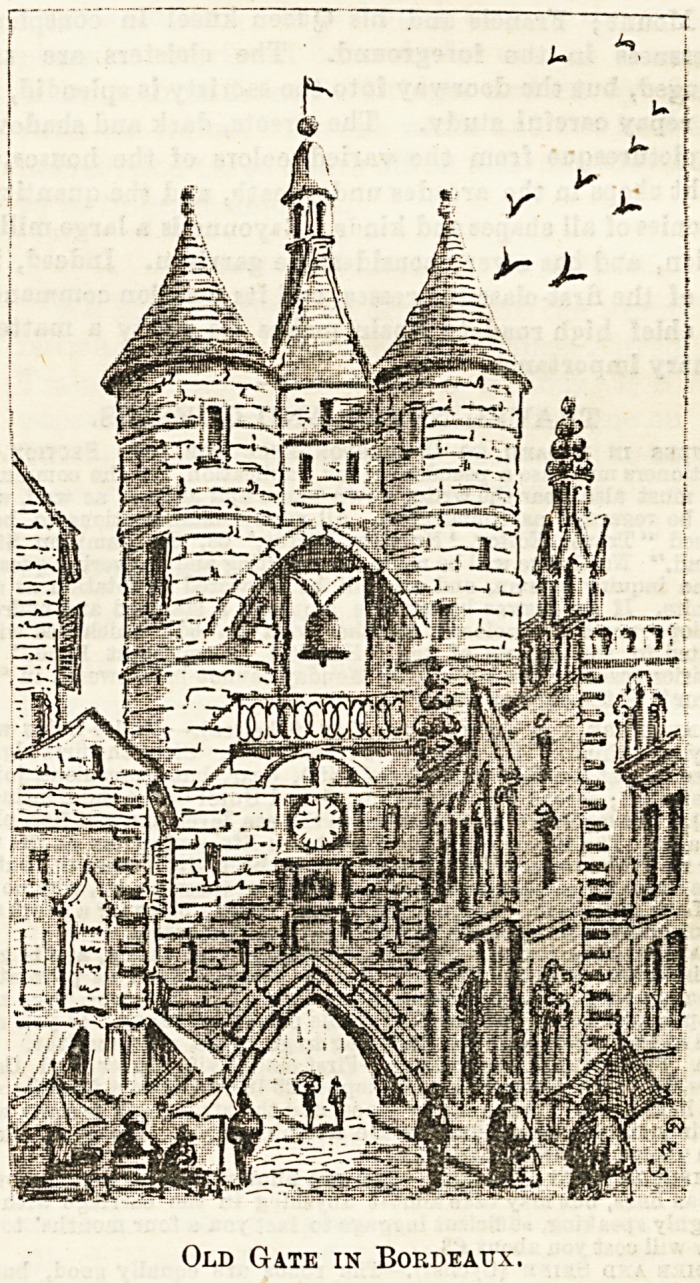# "The Hospital" Nursing Mirror

**Published:** 1899-03-11

**Authors:** 


### "The Hospital" Nursing Mirror.

Being the Nursing Section of "The Hospital."

[Contributions for this Section of "The Hospital" should be addressed to the Editor, The Hospital, 28 & 29, Southampton Street, Strand, London, W.C., and should have the word "Nursing" plainly written in left-hand top corner of the envelope.]

### News from the Nursing World.

### VICE-REGAL VISITS.

Lady Curzon paid a visit to the Calcutta Medical College and Hospital within a very short time of her arrival in India. Accompanied by Colonel Fenn, hon. secretary of the Countess of Dufferin's Fund, Lady Curzon went round the wards of the hospital, and afterwards was driven to the Surnomoyi Hostel, where she was received by the lady superintendent, Miss Taylor. Several of the students were introduced to Her Excellency (who congratulated Miss Pitt on her newly-won gold medal), and also Miss Rachel Cohen, M.B., lately appointed to the charge of the new hospital at Rangoon; Miss Statesbury, on leave from Quetta, where she is in charge of the Dufferin Hospital; and Miss Chite, holder of one of the United Kingdom scholarships. Lady Curzon went on to the Eden Hospital, where she visited the wards, evincing much interest in the various cases.

### AN IMPUDENT IMPOSTURE.

Among the various annoyances to which nurses are exposed one of the most irritating arises from the assumption of their garb by all sorts of people who have nothing to do with nursing at all. It is a thing against which we have no means of protecting ourselves and against which we have no redress, so perhaps when we see servant-maids putting on the cloak and cuffs because they think it looks nice, and ladies in narrow circumstances using them to hide their deficiencies of dress, perhaps the best thing we can do is to keep silence on the principle that "what can't be cured must be endured." The matter, however, is somewhat different when we find the nurses' dress used as a cloak to immorality, or as a means of deception. Of the first we will say nothing—indignation in regard to that matter goes beyond words—but we do protest when we find people advertising for "young ladies" to deliver circulars, the said young ladies "to be dressed as nurses." The sham and the fraud which is involved in any such proceedings will of course be obvious enough to our readers, but we are afraid that there are plenty of people ignorant enough to be taken in by such an advertising trick, and to believe that the fact of receiving a circular about some wretched patent food, or soothing syrup, or what not "from a nurse" is some sort of sanction to its employment. We hope then that people will take our word for it that nurses do not deliver circulars; that when they see "young ladies" "dressed as nurses" doing any such thing the whole affair is a fraud, got up on purpose to delude the public; that such "young ladies," or as we should prefer to call them such young persons, are scraped together by advertisement; and that the costumes by which the impudent imposture is effected are, in the terms of the advertisement, "supplied free."

### NEWS FROM INDIA.

Plague has once more made its appearance in Southern India, but the majority of nurses are returning home now that their term of agreement is ended. Nearly all the nurses sent out by the Nurses' Co-operation are amongst the number. It is said that they find they can make more money at home, and have more comfort. One of the cleverest lady nurses, Miss Eva Bickers, has returned home; Miss Campbell did not remain even three months; Miss Bleaney, whose marriage (to a coffee planter), which was announced to take place this month, but which has now been put off, remains in India; Miss Fry and Miss Scott, whose services (after the great unpleasantness in Calcutta) were dispensed with, have been kept on by the Surgeon-General in Bombay. English plague nurses are getting scarce.

### SUFFOLK NURSING ASSOCIATION.

A good opportunity occurs just now for suitable candidates who desire to be trained as district nurses without personal expense. Four County Council scholarships in the gift of the Suffolk Nursing Association are still to be awarded. The second annual meeting of this association was held recently at the Masonic Hall, Bury St. Edmunds, when Sir Henry C. H. Bunbury presided, and a large number of influential folk were present. Eighteen nurses are engaged in sixteen districts, four of the latter having been organized during the past twelve months. The finances are satisfactory.

### MINNEHAHA MINSTRELS.

The famous Minnehaha Minstrels from Manchester visited Burnley recently in order to give one of their entertainments for the benefit of the District Nursing Association. The Mechanics' Hall was crowded with a fashionable and most appreciative audience, who applauded their efforts with the greatest heartiness. The splendid help that these minstrels have given to charities in and about Manchester mounts up to nearly £14,000, that being the amount raised since they began their good work. The troupe number about eighty, and this last concert realised the handsome sum of £100, which has been handed over to the District Nursing Association. The services of a fourth nurse, whose help has been wanted for some time, have therefore been secured to the town.

### THE LINCOLNSHIRE NURSING ASSOCIATION.

Lord Winchelsea's death has deprived the Lincolnshire Nursing Association of a warm friend and liberal supporter. Lady Winchelsea, in consequence of her bereavement, has been unable to entertain the nurses as usual at Haverholme, and the fifth annual report has been issued without the customary general meeting. The year has been one of growing usefulness, and several changes mark the expansion of the work. The first is the appointment of Miss Glover as lady superintendent and secretary. The Queen Victoria's Jubilee Institute for Nurses, owing to pressure caused by the multiplication of county associations, has been obliged to give up the inspection of the society's nurses. The committee has, however, made a grant of £50 towards the expenses incurred. Miss Glover's appointment necessitated the retirement of Miss Sumpster, the lady who has been the valued secretary of the association since its foundation. Five nurses have left the staff during the year—two left at the expiration of their agreement, two were unsatisfactory and dismissed, whilst one resigned on account of her health. Although the association recommend districts to employ hospital trained nurses whenever possible no fewer than eight candidates last year were trained at Plaistow, the cost of seven of them being defrayed by county council scholarships. The expenditure exceeded the income by £35, and the committee fear that, unless more subscribers come forward, they must encroach upon their reserve fund.

### A LECTURE TO NURSES.

Dr. Tom Robinson, always so delightful a lecturer, lately gave the members of the Trained Nurses' Club a most interesting address upon "The Wit and Wisdom of Sir William Gull." Dr. Robinson spoke with the sincerest admiration of the brilliant qualities of that great man, of his hard work, of his extraordinary teaching capacity, of the time and patience he would devote to his cases, his hatred of humbug and intolerance of ignorance, his powers of diagnosis, and his firm religious faith. Dr. Robinson illustrated his remarks with more than one interesting anecdote, and especially dwelt on Sir William's pioneer advocacy of the "expectant treatment" of disease, and of his strong antipathy to specialism. His audience thoroughly enjoyed Dr. Robinson's lecture, and at its conclusion he presented the club with an excellent portrait of Sir William Gull, beneath which he had written Sir William's own definition of the "aims of life"—"Conceptio Dei; negatio mei; ratio rei," or, to put it into English, a conception of God; forgetfulness of self; and an insight into the relation of all things to each other.

### THE NORTH LONDON NURSING ASSOCIATION.

On the last day of February the annual meeting of the North London Nursing Association was held at the Nurses' Home, 418, Holloway Road. The Bishop of Islington presided. The average number of cases nursed per month for the year was 133, amounting to a total of £1,592. The staff consists of from eight to ten nurses, who have with one exception enjoyed good health. That one is now recovering from an attack of typhoid fever, having been successfully treated in the Great Northern Central Hospital. One lady, who has given her services gratuitously for over ten years, has left the staff. In spite of an increase of 30 per cent. in the work accomplished by the association, the expenditure has remained stationary. The year, as regards extraordinary income, has been an exceptional one; and, as such good fortune is not likely to recur, the committee are most anxious to increase the number of subscribers.

### THE SICKNESS OF THE SAINTS.

The armies of science are mustering strongly in war to the death against the insidious and fatal disease of consumption. If it can be banished that is an irrefutable argument that it should be. It is therefore not astonishing that some enthusiastic souls should desire to spend their lives in working out their own salvation in alleviating the sufferers from this dire affliction. The proposal to form a nursing sisterhood to attend specially on what they term "the sickness of the saints" is quite in accordance with the spirit of certain forms of the Christian faith; whilst that it hails from the land of prompt action over which floats the standard of the Stars and Stripes is only what might have been expected. Acknowledging the motive of this "sisterhood" as good, it is impossible at the same time not to question the wisdom of confining nurses to one kind of sickness and that sickness infectious. Their natural immunity to it must be weakened by constant exposure to its influence; precautions, at first so carefully observed, are more lightly regarded as familiarity, and perchance the weariness that comes from monotony, makes them onerous, whilst minds set in one groove lose their elasticity—a loss as grievous to the patient as to the nurse. To combine to stamp out consumption is excellent, to combine to nurse nothing but consumption is a questionable proceeding.

### SANITARY EDUCATION FOR THE PEOPLE.

The Women's Co-operative Guild has done good work during the past two or three years by taking up some subject of national importance as a winter study for its members, engaging competent lecturers to speak on the various topics, and issuing popular pamphlets for general distribution. Two years ago Poor Law questions were under consideration; while last winter matters affecting public health and the laws which protect and control the community formed the subjects of the lecture courses. The guild is the first body of women to make any such attempt to diffuse general information on questions which vitally affect the well-being of the country, and 68 lectures were given on its behalf last season by Miss Alice Ravenhill in 22 large manufacturing centres. During the present winter Miss Ravenhill has been continuing her work for the guild, and has visited many large towns, chiefly in the northern counties. Attention has been chiefly concentrated on "The Housing of the Working Classes" and "Public Health Laws," but "Water Supply" and "The Nation's Food Supply" have also attracted large audiences, and the facts impressed upon them by this means concerning water and milk-borne diseases, tuberculosis, &c., should greatly aid the spread of knowledge. Miss Ravenhill is giving lectures on "Household Science" to the Toynbee Nursing Guild on the Wednesdays in March.

### SHORT ITEMS.

The next examination of the Medico-Psychological Association will take place on May 1st. Monday, April 3rd, will be the last day on which candidates can enter their names.—Two items afford the Bishop Auckland and District Nursing Association reason for congratulation: the contributions resulting from the Nursing Sunday Collection were doubled, and the foundations of the nurses' home have been laid.—There is a brightly written and interesting article on "Books and Work" in the current number of *Nursing Notes,* which private and district nurses will like to read. It is by Miss Loane, the author of other very helpful contributions to the same paper.—The recommendations of General Sternberg as regards the employment of women nurses in the American army are that the number shall not exceed one nurse for every 100 men in the army; that the pay shall not exceed £10 a-month for ordinary nurses, and £15 a month for superintendent nurses; and that all nurses must be graduates of a training school for nurses.—The concert organised by Mr. Robb Harwood, at St. James's Hall, in aid of the Grosvenor Hospital for Women and Children, Vincent Square, Westminster, has resulted in a profit of £227 to the funds of the institution.

### Hints on the Home Nursing of Sick Children.

By J. D. E. Mortimer, M.B., F.R.C.S., formerly Surgical Registrar, &c., at the Hospital for Sick Children, Great Ormond Street.

(*Continued from page* 233.)

### NIGHT TERRORS—HYSTERIA—CHOREA OR ST. VITUS' DANCE.

### Night Terrors.

After a broken sleep a child may start up excited or frightened in a half-conscious state, sometimes flushed and feverish; this may go on for some hours, but more often quickly subsides. Older children may "walk in their sleep." The attack may be induced by over-excitement, such as that of a children's party, or by a late and indigestible supper; when recurring, there is generally an erroneous system of feeding and chronic constipation. Other causes of disturbance are excess of bed-clothing, want of ventilation, obstruction to breathing (as from post-nasal growths), terrifying stories or pictures, &c. The child should be spoken to in a calm and reassuring tone, and made comfortable by rearranging the bed, sponging the face and hands, relieving thirst, and any other attention that may be needed. Often there is, next morning, no recollection, or only a hazy one, of its troubles, and they should not be alluded to.

Convulsions are most usually seen in improperly-fed, ricketty children about the period of teething, but may occur at any age and from various causes—for instance, organic disease of the brain, uræmia, the onset of an acute fever. There may be warnings, such as twitchings of the face or clenching of the hands. In ordinary cases the child should be put in a warm bath for five minutes and cold applied to the head, afterwards wrapped in blankets and kept in a quiet, cool, well-ventilated room. An enema may be given and the abdomen gently massaged.

A child subject to epilepsy or attacks of giddiness from brain disease should never be left alone, and if able to get about must be kept from dangerous places, such as the fire-place, the edges of cliffs, &c. If about to fall he should be caught under the armpits from behind and gently lowered, the nurse putting her left arm under the head whilst her right hand loosens the collar, and if possible saves the tongue by putting something, such as a pencil, between the teeth. No attempt should be made to "hold the child down," but he should be pulled away from a wall or any furniture against which he may hurt himself, or if in bed should be guarded by pillows. Afterwards he should be allowed to recover gradually, and to sleep if so inclined. The subjects of epilepsy should be much in the open air and have little meat and no stimulants.

### Hysteria.

Even in very young children the "hysterical" temperament may exaggerate or otherwise modify the effects of other diseases, or there may be, as in older patients, purely nervous mimicry (not to be confused with wilful shamming) of paralysis, spinal disease, &c. An observant nurse, who has the great advantage of being continually with the child, may be able to afford the doctor much help in forming a diagnosis of a puzzling case. The symptoms are apt to be variable and inconsistent as compared with those produced by the complaint (or supposed complaint) in children with more healthy nervous systems, and there may be evident excitability, self-consciousness, desire of being noticed, &c. It must be always remembered, however, that no case should be pronounced "hysterical" merely because it is otherwise unaccountable, and that infinitely more harm may be done by taking, say, a case of spinal caries for "hysterical spine," than by the converse mistake.

In dealing with these unfortunate little ones the nurse must carefully avoid fussiness and over-indulgence, but without appearance of harshness, which, by provoking a feeling of resentment and self-pity, will do more harm than good. A pleasant, calm manner and a little good-natured "chaff" will often work wonders, whereas unkind ridicule would have a reverse effect. "Hysteria," in fact, should be regarded as a disorder requiring treatment, not as a fault demanding punishment.

### Chorea.—St. Vitus' Dance.

The nurse will often find that on taking charge of a case her first duty is to calm the excitement and alarm which it has caused in the household, this, of course, reacting on the child and making matters worse. Without affording any grounds for a charge of indifference or want of sympathy, she must maintain her own self-possession, and may confidently assure everyone concerned that the grimaces and twistings, although tiresome and distressing, are of no serious importance, and that the vast majority of cases recover perfectly within a few weeks of the onset. It is better, at all events at first, to take as little notice of the convulsive movements as possible. Any effort on the part of the child to "make herself keep still" will be likely to have an effect just the reverse of that which is intended. Still less should any forcible restraint be attempted. If the convulsions are severe the child should be clothed in warm loose garments and laid on a wide bedstead or on mattresses spread on the floor, so that she may have entire freedom of movement without a possibility of hurting herself or being irritated by disarranged bed-clothing. In milder cases she should still rest a great part of her time, preferably on a couch out of doors. Rooms must be kept quiet and thoroughly ventilated, both by night and day. The visits of friends, especially of other children, and even the company of the child's own brothers and sisters, should, as a rule, be prohibited until convalescence is well established. It is most important to see that a sufficient quantity of nourishing (not stimulating) food is taken. If the child has any difficulty in feeding herself, this should always be done by the nurse, and it is a good plan to take off the attention by telling a story, or some similar method. Nasal or rectal feeding may be needed in severe cases. The action of the bowels must be duly observed. Massage and warm sponging will often do good, and in some lingering cases more active treatment, such as shower baths, galvanism, &c., may be ordered. As soon as there are signs of improvement the child should be encouraged to try and keep herself steady, to practise slowly "Swedish movements," or to perform some simple task, but caution is necessary, and she must not be hurried at the risk of disheartening failures. Care must be taken to avoid for a considerable time all kinds of excitement, and especially any worry or fatigue of the mind, lest a relapse occur. Some quiet country place should be chosen, rather than a noisy seaside resort, if the child can go away for a change.

(*To be continued.*)

### Pension Fund Nurses.

### MISS BURNS' WEDDING GIFT.

We have still another contribution to acknowledge, viz., one from Nurse E. H. Bunnett, of Shaldon Lodge, Stanhope Road, St. Albans. In a short while we shall be able to announce that the address for Miss Burns is completed and on view.

### Invalid Cookery.

Some time has elapsed since the last article appeared in this paper dealing with the diet of an ordinary convalescing case, and as the subject was not exhausted then, I feel sure many of our readers will be glad of fresh hints and wrinkles in providing tasty and dainty meals for invalids recovering from some ordinary illness in which they have lost strength and tone, which have to be rebuilt to the greater part by what food they take.

Remember a patient's senses are very keen after an illness, and a very small thing will drive away the appetite. It can be made or marred as much through the eye as the palate; so be very careful that the tray cloth is spotless, silver bright, all daintily arranged, and not too much of anything. If fish is ordered avoid the oily kinds, such as salmon, bloaters, eels, and mackerel. Whiting is the most easily digested, and plaice is considered by some doctors to be lighter than sole.

As to cutlets, the most easily digested are those cooked in a gourmet boiler or steamed; the juices are then retained and the albumen is not hardened, but this "invalid cutlet" does not appeal to many people—they prefer them grilled or egged and breadcrumbed. If fried à la Française in deep boiling mutton and beef fat, they will be quite wholesome as far as the frying is concerned, but if fried in lard quite the reverse.

Should the patient be a man he is certain to like a chop, which must be cut from a nice tender loin and "done to a turn." Rub it with salad oil an hour before cooking and season with a little peppor. If grilling cannot be accomplished place it on a well-buttered tin, cover it with a buttered paper, and cook in a hot oven for fifteen or twenty minutes.

Try and vary the vegetables as much as possible. Many are most wholesome and nourishing, but if plainly boiled every day they become very monotonous. There are a variety of ways of doing them, and a nicely-dressed vegetable will often be welcomed, when, if only plainly boiled, the patient would turn against it.

Puddings, too, invalid milk puddings pure and simple, are dull and uninteresting if they make a daily appearance on the invalid's tray. The various farinaceous foods can be used in making the most delicious puddings, either as soufflés or with fruit. Simple trifles, also sponge puddings make a change. Jellies are refreshing, but cannot be numbered amongst nourishing diets. If the patient has had a substantial meal of meat and vegetables, jelly would be sufficient for a pudding.

Game is now in season, and if not hung too long makes many a nice dinner for the invalid, and it is better to sometimes have it cold than to recook it and so harden and destroy the flavour.

### Weekly Menus.

Sunday.—Roast chicken, bread sauce, potato croquettes, prune trifle.

Monday.—Clear soup, cold chicken, watercress salad, sponge pudding.

Tuesday.—Filleted sole (white), tomatoes farcie, sweet omelet.

Wednesday.—Mutton cutlets, potato chips, braised celery, tapioca soufflé.

Thursday.—Boned pigeon, artichokes, potato ribbons, banana cream.

Friday.—Souchet of sole, cold pigeon, American salad, lemon jelly.

Saturday.—Bouillon, cream of rabbit, baked tomatos, potato loaves, Queen's pudding.

### Recipes.

Bread Sauce.—This is quite a nourishing addition to a meal, and is nice with fish for those who cannot digest melted butter or richer sauces. Boil half a pint of milk with a small shallot, into which stick a clove. Then add two ounces of bread crumbs that have been rubbed through a sieve and one ounce of butter. Let it boil for twelve minutes. Take out the shallot, put in two tablespoonfuls of cream, season with pepper and salt, boil again for five minutes, and serve.

Prune Trifle.—Well wash half a pound of best prunes and soak them overnight, then put them in a saucepan with sufficient water to cover them, half a teaspoonful of grated lemon-peel, and sugar to taste. Cook till tender, then take them up and set aside till cold. Afterwards split the prunes and take the stones out. Put some of the prunes with a little juice in a small glass, and over them sprinkle thickly some stale sponge cake crumbs. Then add more prunes and crumbs, and over all pour some whipped cream, sweetened, and slightly flavoured with vanilla.

Sponge Pudding.—Put the yolks of two raw eggs in a basin with three or four drops of vanilla and a little grated lemon-peel. Beat them well for five minutes, then add three ounces of castor sugar and beat the mixture for ten more. Whip the whites of eggs stiffly with a pinch of salt. Add these by degrees with two ounces of flour and half a teaspoonful of baking powder. Pour this sponge mixture into well-buttered basin, cover with a buttered paper, and steam the pudding for two hours. Turn out on a hot dish and serve at once.

Fillets of Sole (White).—Take the fillets from a sole and bat them out with a knife dipped in cold water. Sprinkle them with pepper, salt, and lemon juice. Place them in a well-buttered tin, and put three tablespoonfuls of cold water and the juice of half a lemon in the tin. Cover the fillets with buttered paper, and cook in a very moderate oven for fifteen or twenty minutes. Warm a little cream, season it with pepper and salt, and put in it six bearded oysters, let them get warm through but not boil. Dish up the fillets of sole and pour this sauce over them.

Tomatoes Farcie.—If any cold chicken remains from the previous dinner cut the meat from the bones and chop it finely, put it in a basin, season with pepper and salt, and moisten with a little cream. Take two nice ripe tomatoes, cut a small piece off the top of each, and scoop out all the seeds. Fill them with the minced chicken, put the tops on again, place them in a tin, and cover with a buttered paper. Cook them in a moderate oven for about twenty minutes or half an hour. Then dish up on a hot dish, and sprinkle with a little chopped parsley.

Braised Celery.—Well wash a good head of celery and tie it round with a piece of string, put it in a stewpan with cold water, and as soon as it boils take it up and wash again in cold water. Put half an ounce of butter in a stewpan with some sliced vegetables—such as carrot, onion, and turnip—lay the head of celery on these, cover all with a buttered paper and the lid of the stewpan, and fry for fifteen minutes. Add about a pint of hock, and let the celery braise for two hours. Take it up, and, if liked, serve some of the gravy freed from fat and thickened with a little cornflour round it.

Banana Cream.—Bananas being one of the most nutritious fruits, it is suitable in almost any form for invalids. Take four, peel them, and rub them through a sieve. Stir into the purée six sheets of leaf gelatine dissolved in a little hot water, add a few drops of lemon and half an ounce of castor sugar, whip a quarter of a pint of cream till quite stiff, add this to the banana purée, mix lightly, and put into a small fancy mould. When set dip the mould in warm water. Pass a dry cloth over the top and turn out.

Souchet of Sole.—This delicious dish is a welcome change, especially as it combines soup and fish. Put a medium-sized sole into a shallow stewpan, cover it with cold water, and add one onion sliced and a little finely-shredded carrot and parsnip, a small bunch of herbs, such as thyme, parsley, and bayleaf, three peppercorns, and a pinch of salt. Bring to the boil, then let the sole simmer gently for eight minutes. Then take it up and keep hot between two plates. clarify the stock by stirring into one pint of it the slightly whipped whites of two eggs. Bring to the boil, then strain through a soup cloth, reheat it. Put the sole in a deep dish and pour the stock over it.

American Salad.—Take a young and tender cabbage, well wash it in salted water, then shred it as firmly as possible into tiny long shreds, shake all the water from them, and place them on a flat dish. Sprinkle them with pepper, salt, a little salad oil, a few drops of Tarragon vinegar, and two tablespoonfuls of cream. Mix all together with two spoons, and fill high up in a small salad bowl.

Cream of Rabbit.—Remove the back fillets and meat from a fleshy skinned rabbit, pass the meat twice through a mincing machine, and pound well, then rub it through a sieve, to two ounces of this purée, add two raw yolks of eggs, one truffle cut finely, pinch of salt and pepper. Mix well together, then add a quarter of a pint of stiffly-whipped cream, and the whites of two eggs that have been whipped stiffly with a pinch of salt. Lightly butter a small mould or some paper Soufflé cases, three parts fill them with the mixture, and bake in a moderate oven for three-quarters of an hour, or twelve to fifteen minutes.

Potato Loaves.—Cook a pound of potatoes in a very little water, so that they are as dry as possible, strain, rub them through a sieve, mix them with the yolk of one egg, half an ounce of butter, pepper and salt. When cold, roll up with a little flour and form into tiny cottage loaves. Brush over with whole beaten up egg, and bake for fifteen minutes.

### Milk Laboratories.

There is little doubt that the tendency to feed the seriously sick through the medium of food prescriptions is on the increase. Invalid kitchens, where every variety of pre-digested foods, wheys, humanised, and other artificial preparations of milk are kept "in stock" or prepared to order, are slowly becoming established in our midst, and reliable dairies are protecting the invalid from boracic and salicylic saturated milk, not only by guaranteeing pure milk but by conscientiously carrying out their assurance of "no adulteration" of any nature. Sensitive stomachs and infantile digestions reject and resent milk treated by preservatives and "sweeteners," and in the interests of the army of invalids it is to be hoped that active legislation will soon take measures to nip nefarious dairy practices in the bud. In the United States—more especially in New York and Boston—some very interesting milk laboratories have been set up with great success, and have proved most helpful in providing milk "modified" to every possible condition of digestion. These laboratories, opened some five or six years since, have introduced an entirely new system of scientific dietetics. They are carried on under medical supervision—all the cows from whom the milk is derived are under daily and skilled veterinary inspection, and the milk is not only produced under the highest hygienic conditions, but is subject to constant bacteriological examination. Prescription forms issued from the laboratory are furnished to any medical men who apply, or who wish to adopt the "modified milk" system, and the list of those who have done so includes the leading members of the profession. By means of these forms the physician can prescribe the exact formula to which he desires the milk to be "modified," and it is very satisfactory to find that the system of providing such modified milk according to prescription has already been introduced into London. A baby patient has, perhaps, a digestion which cannot cope with the normal albuminoid of milk. A certain proportion of this is eliminated, and thus the "modified" milk is made to suit a digestion defective perhaps only in this one particular. Another infant, or perhaps an invalid under "milk-cure" treatment, has trouble in disposing of the normal amount of sugar found in milk. A duly prescribed quantity of this is abstracted from the milk, with perhaps an entirely satisfactory result. The amount of food necessary for each feeding may be noted on the prescription, in which case the specified quantity is sent to the patient in a sealed tube, each tube representing the proper "meal." A visit to a milk laboratory proves a most interesting expedition to anybody interested in dietetics, for here is foreshadowed the tremendous influence which diet is bound in the future to exert in the treatment of the sick. It would be ridiculous any longer, after experiencing the practical results achieved at these laboratories, to suggest that there are any sick people for whom milk cannot be modified so as to "suit." The hygiene of the dairy is illustrated in its perfection at these milk laboratories, from the vast sterilisers in which cans and receptacles are rendered scientifically aseptic, to the exquisite method whereby a daily supply of freshly prepared milk sugar is obtained. All the milk is sterilised or Pasteurised, and in every case the cream is separated from the milk by centrifugal process. The cream is re-added in varying proportions to suit individual digestions. Distilled water, or lime water made with distilled water, is used for dilution, this being apportioned to the needs of each patient rather than according to a general law of probabilities. In specially hot weather a higher temperature is used in the sterilising process, and it is by such a natural method rather than by the addition of harmful chemicals that this "modified milk" is kept sweet. Many delightful and delicious forms of milk drinks are made constantly at these laboratories, so that average invalids who do not need a prescription for the modification of the milk of their diet can at all times secure a supply of sweet wheys, peptonised milk, and the purest cream from a source which is above suspicion. In London such foods may be obtained from reliable dairies, and the work of preparing milk according to prescription is being undertaken by a few. There is but little doubt that it is a system which has a large future before it.

The number of women who nurse their own infants is becoming less and less—that is to say, among the better classes. The neurotic tendencies of the day, the social and professional conditions which tend more and more to call mothers away from their homes, is leading to a multiplication in the number of artificially-fed babies. Various athletic forms of amusement, and the bicycle especially, has much to answer for in depriving the modern baby of that nourishment which is his infantile due. For there can be no question that violent exercise, such as that of "the wheel" and the golf-links, materially lessens not only the secretion but the quality of a woman's milk. No dairyman would make a profit worth speaking of from cows put through an organised course of gymnastics and athletic exercises. Neither can the nineteenth century infant obtain sufficient nutriment for his growth and needs from an "out-door" mother, who does a bicycling record or an 18-hole golf round "between her baby's meals." The science of the laboratory must then be requisitioned to provide a substitute.

### Presentation.

On the occasion of Miss Mulligan leaving the Wolverhampton Borough Hospital, where she has been matron for the last six years, she was presented by the members of the Health Committee and the hospital staff with an inscribed silver tea service. The Mayor (Mr. Councillor Price Lewis), in making the gift, endorsed the chairman of the committee's testimony as to Miss Mulligan's satisfactory, efficient, and conscientious discharge of her duties, and wished her a long, happy, and prosperous life. Miss Mulligan's appointment to the new isolation hospital at Huddersfield has already been noted in our columns.

### A Book and its Story.

### "WINDYHAUGH."

"Windyhaugh,"* has a particular claim on the interest of the medical world, since it is the work of Margaret J. Todd, M.D., who is at present assistant physician to the Edinburgh Hospital for Women and Children, after having taken her M.D. degree at Brussels four years ago. Miss Todd's career has been one of much interest, but it is with her work, under her pseudonym of Graham Travers—as the sucessful littéraire rather than as the scientist—that we are concerned for the moment, and that she attracts the attention of the reading public.

To the delightful breeziness and naturalness of the style of this tale is in a great measure due the pleasure we receive from its perusal, but there is something more than the charm of the manner in which the book is written; there is a ring of reality in the tale itself which compels our admiration. The scene of the opening chapters of the story is laid in Scotland, and the atmosphere of the story is Scotch. The story takes its name from the house wherein Wilhelmina Galbraith lives her early years. The child's surroundings are little in accord with her own unrestrained imaginative nature. She lived alone with her grandmother. "And their home was a quaint old place, almost worthy of a visit for its own sake in these days of artistic villas. The sun and storm of a century or two had called forth no mellow colouring in the iron-gray walls; but here and there a yellow lichen had woven its dainty web, and ivy and old-fashioned roses clambered about at will, concealing as best they could the neutral tints of the stone. A straggling, tangled old shrubbery flanked the house on the right, and in front the close-mown lawn was sheltered from the road by a little plantation of lime trees. Very cool and inviting these looked to the dusty wayfarer when the sunlight streamed through the boughs, and the shadows played on the lawn; but in cloud or storm their shade amounted to gloom, and a sense of mystery, of a haunting 'beyond,' hung over the whole place. They never seemed to be at rest, those trees, and the sweeping sough through their branches was never still; for the house stood high on a terrace above a great arm of the sea, and night after night Wilhelmina was lulled to sleep by the murmur of wind and wave, or startled into wakefulness by their fury."

In such surroundings, with a strict education from the grandmother on the doctrinal meaning of "faith" and "election by justification," the child's mind awakens. She gauged the limits of the spiritual existence as evinced in the narrowed sphere of a Presbyterian teaching; much of the interest of the story centres round her aspirations, her doubts, her questionings, for the child faced the difficulties of life at an early age. And all this is told in the simplest, most natural manner by the narrator of Wilhelmina's history; in fact, one asks at times how far the heroine is a creation of the imagination, how far a study taken from the life. No less interesting than the description of the daughter is the description of her father—the fascinating, thoughtless, extravagant man of the world, who appears at intervals throughout the tale, a man whose very faults have an attraction.

A change in Wilhelmina's life comes to her on the death of the grandmother, the stern Presbyterian grandmother who had ruled the household at Windyhaugh. The child, now several years older than when we first make her acquaintance, goes to London to live with her father's new wife, a kindly superficial woman lacking in all qualities of common sense, the absence of which qualities soon proves a hindrance to the happiness of any home life. Mr. Galbraith contents himself with a brighter atmosphere abroad, and his wife, assisted by Wilhelmina, drags out a sordid existence in the shabby genteel Bayswater lodging house. Here life is carried on on the hand-to-mouth principle, and a daily struggle for existence is among Wilhelmina's mundane cares. Pitiful scenes these are in which to find the child whose early bringing up had been so screened from care and anxiety, but they were circumstances which Wilhelmina met with a courage and endurance which did much to make of her the noble character which she aspired to become and the depressing depths of social wretchedness only acted as a spur upon her to rise to better things. Wilhelmina Galbraith's is the story of a strong character which was tried in many fires, gaining strength in devious paths, and ever holding the high ideal before her; but for a fuller following of the girl's most interesting individuality we must refer the reader to the book itself. Time goes on; change upon change have come to Wilhelmina; the Bayswater lodging-house exists no longer; the empty, frivolous, albeit affectionate, stepmother is dead; and once more Windyhaugh receives the father and daughter under its picturesque roof, though it is a very different place now to what it was in the days of its former mistress. Mr. Galbraith fills the house with people of his own world, and Wilhelmina rules as its mistress. Then comes her marriage and her separation from her husband—a separation prompted by a too self-analytical nature, and once more we find Wilhelmina in London, working again, full of ambition in that "beyond" for which as a child she had sighed. And now it is into her profession that the girl throws the strength of her will. She will succeed and make a name for herself in science. She has gone through with her studies, and is just about to pass the final examination, when the wheel of fortune turns suddenly round once more as unexpectedly as it had done on previous occasions in the girl's career, and Wilhelmina is called upon to give up her vocation and tend her suffering, erring father, and in the same brave spirit in which she had entered into the arduous duties demanded of her in the following of her profession, so in an equally brave spirit does she cast the aspiration so dear to her aside, and the last months of Mr. Galbraith's pitiful career are alleviated by a sweet and willing presence. Wilhelmina had always been a devoted admirer of her brilliant father. She is called on now to minister to the needs of a man who has given himself to drink. "There was no keeping the skeleton concealed any longer. It was flaunting itself in the light of day. . . . Could he, in the old days of his fine reserve, have seen himself as he was now. . . . It was the irony of the situation which affected her most. If ever a man had seemed equal to any and every occasion that man was her father, and now ——!"

And when death finally releases the suffering man, it is over his grave that Wilhelmina and her husband are once more united. Wilhelmina had been tried in many fires and had come out ennobled and strengthened by them, and we leave her happy. It is but with a passing regret we ask why the girl of many aspirations did not do more—accomplish more?—but in the closing page of Dr. Margaret Todd's story the writer forestalls this passing question, which it is likely will be felt by all who have followed her heroine's career throughout these interesting pages. The writer argues thus of her heroine: "It is true she achieved little as we reckon achievement in these days. She carved no statue, painted no picture, composed no oratorio . . . but when all these things have been excluded, there remains that little art of living which has been open in all ages to the wise and to the simple."

* "Windyhaugh." By Graham Travers (Margaret Todd, M.D.) (London: Blackwood and Sons. 1898.)

### The New Hospital for Women.

### THE NEW NURSES' HOME.

The Bishop of London opened the nurses' home that has just been built for the accommodation of the staff attached to the New Hospital for Women on the 7th inst. The speeches appropriate to such occasions were made beforehand, at the annual meeting which had just been held, and will be found in our account of the meeting in The Hospital. The home is an old-fashioned-looking two-storied house. The windows on the ground floor are furnished in front with green jalousie shutters, fixed back in the day time with an iron catch. The back windows are defended by iron bars. It has a respectable and comfortable appearance, which is not belied by the interior. On the right hand of the small hall are the sister-superintendent's rooms. The cosy sitting-room is divided by a curtained archway from an equally cosy bedroom. A few steps further on there is a corridor running right and left. On turning to the left one first comes upon the nurses' sitting-room. It has—as all the rest of the home—walls tinted in buff and stone colour, whilst a moulding about eighteen inches from the ceiling, from which to hang pictures, separates the two tints. The woodwork throughout the home is painted a soft sage green, and the floors are of wood laid in squares, each square made up of three brick-like blocks. In the sitting-room, however, these are hidden by a bright Oriental carpet. This room is furnished with a couple of tables, several easy chairs, a couch, and a piano—the last-named, however, is only lent for a time. The bedrooms are of good size, and are furnished, in addition to the beds, with a chest of drawers, a hanging closet, a washing cabinet, with one strip of carpet or rug lying at the bedside. The portion of the corridor running to the right of the entrance hall leads to lavatories and cupboards on the right hand, and on the left to a charming kitchen with enclosed dresser, and every convenience, as well as to the staircase. Eight bedrooms, a bath-room, lavatory, &c., are arranged on the first floor, whilst the second floor (for the night nurses) is a reduplication of the lower storey. Above is an attic, well lighted, and cross ventilated, where, beside the water storage tanks, there is plenty of box room. It is a delightful home, and the nurses are well pleased with their new acquisition. A beaten copper plate is ready for fixing bearing the following inscription: "In grateful memory of Emily Pfeiffer, by whose charitable bequest this home was built. 1898."

### Where to Go.

Dowdeswell Galleries.—A very interesting series of water-colours and frescoes, entitled "Picturesque Holland," is being shown here. They are the work of Nico W. Jungmann, who throughout his work depicts characteristic strength of expression in every face. The perfection of detail, as is common to so many Dutch painters, is also a strong feature in this work. There are two very beautiful frescoes, companion pictures, the subject of which is sympathetic and the treatment very good.

### Wants and Workers.

[The attention of correspondents is directed to the fact that "Helps in Sickness and to Health" (Scientific Press, 28 & 29, Southampton Street, Strand, London, W.C.) will enable them promptly to find the most suitable accommodation for difficult or special cases.]

Mrs. Monro Allan, Flaler, N.B., would feel obliged to any reader of The Hospital if they would kindly give her any information regarding the working of district nurses' associations, especially if they have been on their committees, and how it works.

### The Society for the Protection of Birds.

A numerous and interested audience assembled at the Westminster Palace Hotel on February 28th to receive the eighth annual report of the Society for the Protection of Birds. Sir Edward Grey, Bart., M.P., took the chair, and after some preliminary remarks upon the progress and objects of the society by Mr. Montagu Sharpe, chairman of the Executive Committee, moved the adoption of the report. This was seconded by Mrs. Creighton, and carried unanimously. The office of the society, which now numbers 20,000 members, has been removed to 3, Hanover Square. Branches have been formed in various parts of England and abroad. Many methods are in vogue for the protection from extermination of our native birds. The county councils are taking an interest in the matter. That of Middlesex has obtained a consolidation order prohibiting the killing and snaring of birds on Sundays. All the speakers denounced the practice of wearing egrets, and other feathers necessitating the slaughter of birds for adornment only. Plovers' eggs are henceforth to be protected, and Mrs. Creighton trusted that larks would soon also be banished from the table. The income of the society was £734.

### Minor Appointments.

Leyton Isolation Hospital.—Miss Gertrude Appleford was appointed Matron here on February 24th. She was trained by and attached to the staff of St. John's House, Norfolk Street, Strand, from 1888 to 1893. Her subsequent appointments have been as follows: Charge nurse at the North-Eastern Fever Hospital for two and a-half years; charge nurse at the Park Hospital, Hither Green, S.E., where she was also in charge of the nurses' home and night superintendent.

Warrington Infirmary.—Miss May Rose, who has had one year's training at Walsall Hospital, and three years' at Stockport Infirmary, has been appointed Charge Nurse here. Miss Rose has also had some experience as district nurse at Stockport.

General Hospital, Newark.—Miss Minnie Walker has been appointed Charge Nurse of this hospital. She was trained for three and a-half years at Chesterfield and North Derbyshire Hospital, and has been staff nurse for eighteen months at the Royal Infirmary, Sheffield.

London Temperance Hospital.—On February 15th Miss E. M. Fitch, who was trained at the Royal Isle of Wight Infirmary and County Hospital, Ryde, was appointed Staff Nurse at the above hospital.

North Riding Infirmary, Middlesbrough.—Miss Marie Jones, who was trained at the Rochdale Infirmary, has been appointed Sister of the male accident wards of the above institution.

### BOOKS RECEIVED.

Swan Sonnenschein and Co.

"A Century of Vaccination." By W. Scott Tebb, M.A., M.D. (Cantab.), D.P.H.

J. and A. Churchill.

"A Code of Rules for the Prevention of Infectious and Contagious Disease in Schools." Issued by the Medical Officers of Schools Association.

Gardner, Darton, and Co.

"Parish Problems; or a Word with Everyone on the Parish Councils Act." By Lady Baker.

Welford and Sons.

"Modern Dairy Sanitation."

### Everybody's Opinion.

[Correspondence on all subjects is invited, but we cannot in any way be responsible for the opinions expressed by our correspondents. No communication can be entertained if the name and address of the correspondent is not given, as a guarantee of good faith but not necessarily for publication, or unless one side of the paper only is written on.]

### HOMES FOR THE AGED.

"A HOSPITAL SISTER" writes: I see an inquiry in your paper this week for "Homes for the Aged." A few years ago I had an old man (patient) under my care; he was homeless, but had a little money. When he was ready to leave the hospital I was very worried about him, as he was over eighty years of age and needed some attention. He was a Roman Catholic, so I consulted the parish priest, and he advised his removal to a little convent home, nursed and managed by the "Little Sisters of the Poor." I took my patient and saw the home for myself. The sisters seemed to me to be brimming over with gentleness and kindly feeling, the home spotlessly clean, the little infirmary just an "abode of peace." I am not a Roman Catholic myself, but when I saw that little convent I thought I had never seen such a restful, quiet place before. I inquired if all the patients were Roman Catholics, and found that they were not, some of them being Methodists. All patients must be over sixty years of age on admission. If they can afford to pay, the money is very acceptable, if not they are provided for. I have no doubt that preference is given to Roman Catholics. There are many homes scattered about the country, and all worked by the same sisterhood. If you consider the little information of any use I should be pleased if you would insert it in your paper.

### A Simple Method of Pasteurising Milk.

THE fact that Pasteurisation is far more useful to the general public than sterilisation is becoming rapidly recognised. An absolutely sterile milk has lost many of its nutritious qualities, and is less suitable for the sustenance of very young children than milk which has not been subjected to a very high temperature. But the point arises that raw milk is an active agent in the dissemination of disease, and this at every cost must be guarded against. Pasteurisation appears to meet the needs of the case in every way, and where there is only a comparatively small quantity, sufficient for the daily use of an average hospital or institution, the apparatus described in the following paragraph is simple to manage, effectual, and inexpensive. Two covered tinned steel pans are procured, one being smaller than the other to allow of the larger holding the smaller, and also sufficient water to surround the smaller pan to within a few inches of the top. The outer pan should be copper bottomed, and under this a round gas coil should be fixed. Boiling water is placed in the outer pan and the milk to be treated in the inner. By means of the gas, fire, and hot water the temperature of the milk is raised to 160 deg. Fahr. as rapidly as possible, and this temperature is maintained for from 30 to 40 minutes, and immediately the milk is cooled by means of a refrigerator. By this process the germs of typhoid, scarlet fever, and diphtheria are destroyed, and as the degree of heat employed renders the bacillus lactici acidi (which causes souring) inactive, the keeping properties of the milk are much increased without the corresponding loss of nutrients which would have taken place had the milk been rendered absolutely sterile. This apparatus has the advantage of being easily adapted to the making of Devonshire cream in small quantities, which is a useful substitute for the unpalatable cod-liver oil.

### For Reading to the Sick.

### HEALTH IN JESUS.

IF Himself He come to thee, and stand
Beside thee, gazing down on thee with eyes
That smile and suffer; that will smile thy heart,
With their own pity, to a passionate peace;
And reach to thee Himself the Holy Cup,
Pallid and Royal, saying, "Drink with Me!"
Wilt thou refuse? Nay, not for Paradise!
The pale Brow will compel thee, the pure Hands
Will minister unto thee; thou shalt take
Of this Communion through the solemn depths
Of the dark waters of thine agony,
With heart that praises Him, that yearns to Him,
The closer for that hour. Hold fast His Hand
Though the nails pierce thine too! Take only care
Lest one drop of the sacramental wine
Be spilled, of that which ever shall unite
Thee, soul and body, to the Living Lord.
—*H. Hamilton-King.*

God gives us light and love, and all good things
Richly for joy and power, to use aright:
But then we may forget Him in His gifts—
We cannot well forget the hand that holds
And pierces us, and will not let it go,
However much we strive from under it.
The heavy pressure of a constant pain,
Is it not God's own very finger tips
Laid on thee in a tender steadfastness?
—*H. Hamilton-King.*

I thank Thee more that all our joy
Is touched with pain;
That shadows fall on brightest hours,
That thorns remain;
So that earth's bliss may be our guide
And not our chain. —*A. Proctor.*

### Reading.

And the whole multitude sought to touch Him; for there went virtue out of Him, and healed them all.—*Luke* vi. 19.

There is health in Jesus. He came from heaven with all the health of heaven in Him; health, like sunshine, flowing out irrepressibly; health of every kind; health without measure; health inexhaustible. The balm of the mountains of Gilead might wither down and die out; this heavenly balm could not. It was like the leaves of the tree of life, never failing, ever growing, and ever green. The physicians of Gilead died till none was left; this Physician dies not. He is the everlasting Christ, the Son of God. All health, and skill, and kindness are to be found in Him; for not only is He perfect man but very God; nay, and the fulness of the healing Spirit without measure dwells in Him. . . . On every side we may approach Him. At any time, and in any way, we may come. Whatever be the length or the deadliness of our disease, we may come. The depths of divine compassion are infinite. So are its heights. God's pitying love takes in the worst sinner that ever breathed the air of earth. Wide as earth, wide as heaven, wide as His own infinite heart, such is the pitying love of God.—*Bonar.*

### OUR CONVALESCENT FUND.

OUR Convalescent Fund is the richer this week by Nurse Jessie M.'s gift of 2s. 6d. We would again remind our readers that it takes many half-crowns to send a sick nurse for a holiday.

### Travel Notes.

By Our Travelling Correspondent.

### XIII.—ARCACHON, BORDEAUX, AND BAYONNE.

Whilst in or near Pau there are a few places of interest that should be visited, firstly Arcachon, which is for some cases a most desirable health resort. It is situated about 30 miles from Bordeaux, and possesses a very sedative climate peculiarly well suited to some nervous affections; moreover, it is warmer in winter and cooler in summer than other watering places of about the same position. Almost all chest troubles and throat affections are benefited by a residence there. Doubtless the enormous pine forests exercise a salutary effect; there are two distinct seasons, a summer one from July to September, and a winter from November to May; indeed the whole year Arcachon may be said to be full.

### Amusements.

It is not so rich in these sort of things as Biarritz, but still it is by no means dull, and there is a good deal of friendly visiting among the English quite different from the gay Biarritz, where the attitude is not nearly so neighbourly, and where unless you have your own circle, or good introductions, you will observe amongst the other sojourners in the gay little town a tendency to the position of stately isolation, so often affected by the travelling Briton. "Don't presume to speak to me or it will be the worse for you" is so often the apparent state of our minds on the Continent; and a great pity it is, for really we can be quite pleasant when we unbend, and if misfortune or illness should overtake us how all this stupid reserve is cast to the winds, and all are engaged in a friendly rivalry as to who can do the most for us. There are two parts to the town of Arcachon devoted to the summer and winter residence. That portion called the Ville d'Hiver is built among the pines, and is sheltered from the cold winds. Here is the Casino, which contains a good restaurant, reading and concert rooms, and there are a fair number of balls given. The grounds are beautiful, and a great comfort to those who cannot walk far. Then there is boating and ideal bathing, which is managed not from bathing-machines, but from little sentry-boxes, fixtures under the lee of the houses built on the shore. It is very easy to run up and down to Bordeaux; the journey only occupies an hour.

### A Short Journey from England.

One great advantage enjoyed by Arcachon is that the journey is comparatively short. You leave Charing Cross at eleven in the morning, and are at Arcachon (if you travel straight through) in time for breakfast the next day. Fares, first class, £5 8s. 7d.; second class, £3 14s. 10d.

### Bordeaux.

Interesting as Bordeaux is in many respects, it is hardly a place to stay in, but you will very probably rest a night, or perhaps two, on your way to Pau, and thus be able to see all the principal points of interest, and should you be resident at Arcachon little excursions for the day to Bordeaux will be one of your amusements. It is, as most people are aware, situated on the Garonne, and its position is magnificent, the river is immensely wide, and full of busy shipping. The principal industry is that connected with the wine trade, and signs of great commercial prosperity are everywhere apparent. It is very interesting to walk along the Quartier des Chartrons, close to the Place des Quinconces, where is the focus of the wine trade. By far the larger quantity of Vins de Bordeaux, which we call claret, is shipped to England.

### The Chief Places of Interest.

There is still a fragment of what must have been a very large amphitheatre at the time when Bordeaux was the Roman city Burdigala; there are only remaining six arches, but it is estimated that it must have held 1,500 people. The Cathedral is well worth a visit. It is supposed to have been built by the English during their possession of the city. There was an earthquake in the fifteenth century which did much damage, and the restorations necessary were not well carried out. Our Richard II. was baptized here, and Anne of Austria's wedding was also celebrated here in 1615. Those interested in ecclesiastical architecture will like to study the Romanesque Church of St. Seurin; the miserere seats are very fine, and the bishop's throne is a mass of rich sculpture. There are two other churches both worth seeing—St. Michel and St. Croix, the latter of the twelfth century. There are very pleasant excursions up and down the

Old Gate in Bordeaux.

Garonne daily, and a ferry puts you across the river perpetually.

### Bayonne.

It is an easy day's excursion from Pau to Bayonne and back, and though there is much of interest for the artist, photographer, and antiquary, I should by no means recommend a stay there. It is a very dull town, and the houses, especially in the older quarter, immensely high and crowded together. The city partakes a little of a Spanish character, as shown by the multitudes of iron balconies. One gazes with great interest at the older quarters and the ramparts, and thinks with unfailing interest of the tremendous siege in 1814 by the allies. In those days the wonderful bridge over the Garonne was non-existent, and our troops performed the marvellous feat of throwing across a bridge of boats in the face of the enemy, and in defiance of the natural difficulties presented by the terrible Bar, which is passable only at high water.

OUR SOLDIERS' GRAVES.

I think every English man or woman must feel interested in the two cemeteries, where are the graves of some twenty officers who fell in that protracted siege. They are chiefly those who perished in the cowardly sortie made by the garrison after a truce was proclaimed, and the English, therefore, were totally unprepared for an assault. The Coldstream Guards are buried in the little cemetery the nearest to the Adour, and the Fusiliers a mile or so to the east.

THE CATHEDRAL.

The first thing the sacristen points out to you in this noble church is the window presented by Francis I., as a thank-offering after his captivity in Spain after the battle of Pavia. It is well worth observing—the subject is the Sermon on the Mount; Francis and his Queen kneel in conspicuous artlessness in the foreground. The cloisters are sadly damaged, but the doorway into the sacristy is splendid, and will repay careful study. The streets, dark and shadowed, are picturesque from the varied colors of the houses, the bright shops in the arcades underneath, and the quantity of balconies of all shapes and kinds. Bayonne is a large military station, and has a very considerable garrison. Indeed, it is one of the first-class fortresses, and its position commanding the chief high road to Spain makes its safety a matter of primary importance.

### TRAVEL NOTES AND QUERIES.

RULES IN REGARD TO CORRESPONDENCE FOR THIS SECTION.—All questioners must use a pseudonym for publication, but the communication must also bear the writer's own name and address as well, which will be regarded as confidential. All such communications to be addressed "Travel Editor, 'Nursing Mirror,' 28, Southampton Street, Strand." No charge will be made for inserting and answering questions in the inquiry column, and all will be answered in rotation as space permits. If an answer by letter is required, a stamped and addressed envelope must be enclosed, together with 2s. 6d., which fee will be devoted to the objects of the "Hospital Convalescent Fund." Any inquiries reaching the office after Monday cannot be answered in "The Mirror" of the current week.

SOMEWHERE FOR APRIL AND MAY (Chelsea).—Aix-les-Bains would suit you, I think. It is four hours from Lyons. Sleep the first night at Marseilles, at the Terminus Hotel (lift), dear, but convenient, joining the station; second night at Lyons, Grand Hotel Collet and Continental (lift). Aix-les-Bains is 850 feet above the sea level, and not hot, plenty of walks. The Grand Hotel du Nord has a lift, and rooms on the third floor would be reasonable. Annecy would be a little cheaper, but not quite so suitable. If you do not like either of these places, why not try the Lake of Geneva. Avignon is an ideal resting place for a short stay, full of interest, but you might find it perhaps too warm.

MALAGA (Khartoum).—It is very mild and also sunny, and is growing in favour with the English but its distance from us prevents its becoming so popular as the Riviera. Living is, however, very much cheaper, on account of the favourable money exchange. If you are a good sailor, it would be less fatiguing to go by sea viâ Gibraltar.

LA BOURBOULE (Carnation).—First class, viâ Dover and Calais, £5 0s. 10d.; viâ Newhaven and Dieppe, £3 18s. 3d. Apart from its value as a health resort it is a lovely place, and the strong and healthy would find innumerable excursions in the neighbourhood. Living is, like all such places, somewhat expensive.

THROUGH ITALY (Puzzled).—You are allowed no free luggage on the Italian lines, but may take almost anything in the carriage with you. Roughly speaking, sufficient luggage to last you a four months' tour in Italy will cost you about £3.

LOIRE AND SEINE (Cyclist).—The roads are equally good, but the Loire is the more interesting trip, its banks being as thickly planted with castles as the Rhine. Expense rather greater on the Loire, as the distance is more and there is more ground to cover by the river banks.

HOLLAND (Amphibious).—Yes, it is rather expensive, partly because the country is not so well known to tourists, and therefore competition is slack, and partly because the money exchange is not so advantageous. Tell me where you want to go, and I will advise you further.

*For Travel Advertisements see page* xvii.

### Appointments.

GRIMSBY AND DISTRICT HOSPITAL.—On February 7th Miss Annie S. Longstaffe was appointed Matron of the above institution. She was trained, and afterwards for three years at, the North Staffordshire Infirmary Eye Hospital, where she was sister-in-charge of the various wards of the operating theatre. The next appointment was at the Wolverton General Hospital, where she remained two years, and where for part of that time she was deputy matron.

### Notes and Queries.

**The contents of the Editor's Letter-box have now reached such unwieldy proportions that it has become necessary to establish a hard and fast rule regarding Answers to Correspondents. In future, all questions requiring replies will continue to be answered in this column without any fee. If an answer is required by letter, a fee of half-a-crown must be enclosed with the note containing the enquiry. We are always pleased to help our numerous correspondents to the fullest extent, and we can trust them to sympathise in the overwhelming amount of writing which makes the new rules a necessity.**

**Every communication must be accompanied by the writer's name and address, otherwise it will receive no attention.**

*Training.*

(238) Can you kindly tell me whether, after two or three years' training in a provincial hospital, say, the Kent and Canterbury, I should be eligible to enter the Army Nursing Service or to receive an appointment in any hospital abroad? 2. Can you also tell me if a nurse trained in a provincial hospital takes in any case so good a standing as one trained in a London hospital?—*A. E.*

The regulation qualifying candidates for the Army Nursing Service in this respect is that she must "have had at least three years' preliminary training and service combined in a civil general hospital." 2. The comparative merits of the various training schools depend entirely upon the standard from which they are judged. As good work is done in the provinces as in London, but it should be remembered that as the larger institutions afford more ample facilities for study and experience (though the attention bestowed on the individual may be less than in the smaller ones), so their reputation often carries greater weight in securing appointments for the nurses trained in them.

*Blushing.*

(239) Some time ago a lady patient of mine asked me for a cure for blushing. She is 29 years of age, and now perfectly healthy. But when she meets a person of her acquaintance her face becomes perfectly crimson. Patient has tried several remedies without success, and is very unhappy. Could any nurse help her?—*Nurse Nina.*

The patient should lead a regular and healthy life, eating, sleeping, and resting rightly and regularly. She should wear loose clothing, and make up her mind that a blush more or less is neither here nor there, and that no one cares twopence about it but herself. In course of time she will probably forget the habit, and discover herself cured. Beware, however, of nostrums.

*Open-air Treatment.*

(240) Can you please tell me if there is a home or hospital in England where open-air treatment is used for consumption?—*Nurse A.*

The National Hospital for Consumption, Ventnor, the National Sanatorium at Bournemouth, the Cromer Experimental Sanatorium, the Victoria Hospital for Consumption at Craigleith. Dr. Rufenacht Walters' recent work on Sanatoria for Consumptives mentions the following British Sanatoria for Paying Patients: Pool Road Sanatorium, and Sunny Mount Sanatorium, Bournemouth; Denver Sanatorium, Denver, Norfolk; Cotswold Sanatorium, Cotswold Hills; Mundesley Sanatorium, Mundesley, Norfolk; Ringwood Sanitorium, Ringwood, Hants.

*India.*

(241) Can you kindly tell me to whom I should apply for particulars as to the Up-country Nursing Association in India? (2) also, if there are other associations in England for supplying nurses for India?—*G. E.*

Major-General Bonus, The Cedars, Strawberry Hill, is the Hon. Secretary of the Up-country Nursing Association. 2. No; not for India only.

*Nurse-Matron.*

(242) May I draw your attention to the enclosed advertisement which appears in your issue of December 31st (see No. 2,501). In the name of the Nursing profession I should like to ask why a woman, whose only stated requirement is "to understand laundry work," should be styled a nurse-matron? I should also like to know what kind of members compose the Kingsthorpe Urban District Council.

In advertising for a nurse-matron the members of the Kingsthorpe Urban District Council doubtless presumed that only applicants possessing the necessary nursing qualifications would apply. But as knowledge of laundry work is not part of a nurse-matron's equipment, it was probably thought essential that this requirement should be specially mentioned.

*Male Nurse.*

(243) Which is the best newspaper agency through which male nurses can get cases in London? (2) Who would you recommend as a teacher of massage?—*J. D. B.*

You will probably find that our advertisement columns are the best medium for obtaining nursing work. You will also find in them the addresses of several well-known teachers of massage.

*Epileptic Child.*

(244) I have a little boy, aged seven, subject to epileptic fits, sometimes seven or eight a day, and finding him such a great care at home, and it being bad for the other children to see him in such a state, I am anxious to know what to do with him, whether to put him in a doctor's family or a home about which at the present time I know nothing. I may add my means will not allow of great expense.—*Jack.*

It is impossible to advise without a full knowledge of all the circumstances of the case. We would recommend to your notice, before you make any decision in the matter, the excellent results obtained from the system pursued at the colony at Chalfont St. Peter's by the National Society for the Employment of Epileptics (office, 12, Buckingham Street, Strand, W.C.). The only other home for male epileptics in England is at Maghull, of which the office address is 3, Dale Street, Liverpool.

## Figures and Tables

**Figure f1:**